# Economic evaluation plan of a randomised controlled trial of intra-nodular injection of anti-TNF and placebo among patients with early Dupuytren’s disease: Repurposing Anti-TNF for Treating Dupuytren’s Disease (RIDD)

**DOI:** 10.12688/wellcomeopenres.14936.2

**Published:** 2019-02-12

**Authors:** May Ee Png, Melina Dritsaki, Alastair Gray, Rafael Pinedo-Villanueva, Oliver Rivero-Arias, Jagdeep Nanchahal

**Affiliations:** 1 Nuffield Department of Orthopaedics, Rheumatology and Musculoskeletal Sciences, University of Oxford, Oxford, OX3 7LF, UK; 2 Health Economics Research Centre, Nuffield Department of Population Health, University of Oxford, Oxford, OX3 7LF, UK; 3 National Perinatal Epidemiology Unit, Nuffield Department of Population Health, University of Oxford, Oxford, OX3 7LF, UK; 4 Kennedy Institute of Rheumatology, University of Oxford, Oxford, OX3 7FY, UK

**Keywords:** dupuytren, anti-TNF, adalimumab, economic, cost-effectiveness

## Abstract

Dupuytren’s disease (DD) is a common fibroproliferative condition of the palmar and digital fascia of the hand; however, there is currently no approved treatment for early stage DD. The objective of this paper is to describe the methods applied to assess the cost-effectiveness of adalimumab injections compared to usual care for controlling the progression of early stage DD in the Repurposing Anti-TNF for Treating Dupuytren’s Disease (RIDD) trial.

Measure of effectiveness and resource use will be obtained from a randomised clinical trial, carried out in three healthcare centres, and recruiting a minimum of 138 patients aged 18 years and above with a diagnosis of early stage DD. Resource use and utility measures (quality-adjusted life years) will be collected at 3, 6, 9, 12 (primary outcome endpoint) and 18 months post-randomisation. A within-trial cost-utility analysis (CUA) will be conducted at 12 months and if the intervention is effective, a decision analytic model will be applied to estimate the lifetime effectiveness and costs. The analysis will be performed from a health system (National Health Service and personal social services) perspective. Sensitivity analysis will be conducted to assess the robustness of the results.

RIDD is the first randomised controlled trial with an economic evaluation conducted among patients with early stage DD. The protocol described here records our intent to conduct both a within-trial CUA alongside the RIDD study and a lifetime CUA using decision-analytic modelling.

## Introduction

Dupuytren’s disease (DD) is a common and progressive fibroproliferative disorder of the palmar and digital fascia of the hand that affects 0.6% of the general population aged 18 years and 12% among those aged 55 years in Western countries ^1^.

Current treatment for DD, which includes fasciectomy, needle fasciotomy and collagenase fasciotomy, aims to correct the flexion deformities and restore hand function and is recommended when the digital flexion contractures limit hand function and/or the proximal interphalangeal joint is flexed to 30 degrees or more. Ideally, treatment of DD would be directed towards patients with early stage disease, defined as flexion deformities of 30 degrees or less at the metacarpophalangeal and/or at the proximal interphalangeal joint (with a maximum total flexion deformity of 60 degrees), in order to prevent progression of cords development and flexion contractures of the digits. However, there is currently no approved therapy for the treatment of early stage DD and a recent systematic review suggested a lack of robust evidence for treatments, including radiotherapy and intra-nodular steroid injection, proposed for this group of patients ^2^.

No previous study reporting economic evaluations of interventions for patients with early stage DD has been identified ^3^. However, a recent systematic review published by the trial team ^3^ identified four studies, all of which synthesised evidence from various sources, rather than a single randomised controlled trial, and reported the cost-effectiveness of collagenase clostridium histolyticum injection, percutaneous needle fasciotomy or limited fasciectomy among patients with late stage DD.

Here, we present a summary of a health economics analysis plan being undertaken to the second part of the Repurposing anti-TNF for treating Dupuytren’s disease (RIDD) trial, which targets patients with early stage DD. A detailed description of the study design is available in the published protocol which contains details on the methodology (e.g. recruitment, interventions, approval/consent, etc.) ^4^. This trial has been registered with the European Clinical Trials Database (EudraCT: 2015-001780-40) and its Ethics Reference is 15/SC/0259. In brief, a minimum of 138 participants aged 18 years old and above, with early stage DD will be recruited from two UK centres and one centre from the Netherlands. Participants will be randomised to receive either anti-TNF (adalimumab) or placebo (normal saline) injection in a 1:1 ratio. The optimal dose and formulation of adalimumab was found to be 40 mg in 0.4 ml in our dose-ranging phase 2a clinical trial ^5^. The primary objective of the second part of the trial is to determine if optimal dose of anti-TNF injection is superior to placebo injection in controlling disease progression among patients with early stage DD by assessment of nodule hardness at 12 months after the first treatment. Secondary objectives include comparing the development of Dupuytren’s nodules and its associated cord, flexion deformities of the fingers and impairment of hand function for participants between each treatment arms and monitoring for adverse events ^4^.

## Methods

The objectives of the economic evaluation conducted in this study are to assess the cost-effectiveness of anti-TNF compared to usual care (i.e. no treatment) among patients with early stage DD via a within-trial cost-utility analysis (CUA), and to model lifetime cost-utility if anti-TNF is shown to be effective in controlling disease progression at 12 months follow-up (primary outcome end point). No treatment instead of placebo will be used as the comparator. In the RIDD trial, placebo is being administered as an experimental control and not used in routine care for patients with early stage DD.

### Study design

The first proposed economic evaluation involves conducting a CUA of anti-TNF compared with usual care (no treatment) using quality-adjusted life years (QALYs) gained as the main health outcome measure alongside the clinical trial at 12 months. The second proposed economic evaluation consists of using decision-analytic modelling to perform a lifetime CUA based on RIDD trial results and additional data from published literature for predicted lifetime QALYs and healthcare costs.

For both analyses, a health system (i.e. National Health Service (NHS) and personal social services (PSS)) perspective will be adopted for the base case analysis, where the economic evaluation is conducted with the most likely or preferred set of assumptions and values, as recommended by the National Institute of Health and Clinical Excellence (NICE) ^6^, while the societal perspective will be examined as part of the sensitivity analysis.

### Estimation of costs

***Direct medical costs related to trial.*** As we assume that there are no significant differences in direct medical cost between trial arms other than the drug being administered, all direct medical cost related to the trial except manpower is excluded from the cost estimation. Since the usual care is no treatment, the cost of manpower will be included for the anti-TNF group only. Manpower cost will be estimated by assuming that the injection will be administered by a medically qualified clinician at consultant level in the outpatient setting.

The type of injection (adalimumab or saline), the estimated volume administered in case of partial administration as well as any optional application of local anaesthetic cream will be recorded in the trial case report forms (CRFs). In the case of anaesthetic cream application, a dosage of 1500 mg will be assumed ^7^. Potential adverse events at the injection site (local itching, redness, blister, nerve injury, local bruising, and haematoma) will be captured in the CRF. Unit cost of the injection will be obtained from the latest version of the British National Formulary (BNF) ^8^ ( Table 1).

**Table 1.** Resource items utilised in RIDD trial.

Resource item	Unit	Source	Reference to source
**Direct medical costs related to trial (drug administration)**			
Success of injection (fully)	ml	BNF	[ 8]
Success of injection (partial)	ml	BNF	[ 8]
Anaesthetic cream	mg	BNF	[ 8]
**Other direct medical costs**			
Inpatient care	visit	NHS Reference cost	[ 9]
Outpatient care			
Hand surgery: Surgeon consultation	visit	NHS Reference cost	[ 9]
Hand surgery: Radiotherapy	visit	NHS Reference cost	[ 9]
Hand surgery: Steroid/collagenase injection	visit	NHS Reference cost	[ 9]
Hand surgery: Dressing change	visit	NHS Reference cost	[ 9]
Radiology: Ultrasound scan	visit	NHS Reference cost	[ 9]
Physio- or hand therapy	visit	NHS Reference cost	[ 9]
Emergency department	visit	NHS Reference cost	[ 9]
Primary and community care			
General Practitioner	visit	PSSRU	[ 10]
General Practitioner	home visit	PSSRU	[ 10]
General Practitioner	phone call	PSSRU	[ 10]
Practice nurse	hour	PSSRU	[ 10]
Physiotherapist	hour	PSSRU	[ 10]
Occupational therapist	hour	PSSRU	[ 10]
Calls to NHS 111	hour	PSSRU	[ 10]
Medication		BNF	[ 8]
**Direct nonmedical cost**			
Personal social services			
Meals on wheels	day	PSSRU	[ 10]
Laundry services	load	North Yorkshire County Council	[ 11]
Social worker	visit	PSSRU	[ 10]
Care worker/ help at home	visit	PSSRU	[ 10]
Missed work		RIDD trial	
Travel		RIDD trial	
Child care		RIDD trial	
Help with housework		RIDD trial	
**Indirect cost**			
Income lost		RIDD trial	

BNF, British National Formulary; NHS, National Health Service; PSSRU, Personal Social Service Research Unit.

***Other direct medical costs.*** Utilisation of health and social care services that are not related to the trial will be collected through a patient-completed questionnaire at 3, 6, 9, 12 and 18 months after the baseline injection. The questionnaires will capture the frequency of use of community-based health and social care services, number and duration of admissions to inpatient wards, number of diagnostic tests (ultrasound scan), use of outpatient services (physiotherapy, emergency department, surgeon consultation, radiotherapy, dressing change) and medication use for the past three months at each follow-up time points. Unit costs for each resource item will be sourced from the latest available national sources, e.g. NHS Reference cost and personal social service research unit (PSSRU) ^9, 10^. The defined daily dose (DDD) of each trial-related medication will be obtained from the World Health Organisation (WHO) website using the relevant anatomical therapeutic chemical (ATC) classification code ^12^. Unit costs of these medication will be obtained from the BNF ( Table 1).

***Direct nonmedical cost and indirect cost.*** Data collected in the participant questionnaires at each time point will also capture the direct non-medical (personal social services, travel expenses, cost of childcare and help with housework) and indirect costs (income loss) borne by participants and carers as a result of their health state. Unit costs will be obtained from national sources or patients ( Table 1).

### Estimation of health utilities

Impact on participants’ health-related quality of life in each arm will be assessed using data from the EQ-5D-5L (five-level version) instrument collected at baseline, 3, 6, 9, 12 and 18 months from baseline ^13^. As per the NICE position statement, responses to the EQ-5D-5L will be converted into multi-attribute utility scores using an approved “cross-walk” to the three-level instrument and its established utility algorithm for the UK, using the mapping function developed by van Hout *et al.* ^14^.

The responsiveness of the EQ-5D-5L instrument in early stage DD has not been evaluated and hence the quality of life impact according to number and severity of affected fingers will also be assessed based on utilities from a recent discrete choice experiment using responses from the UK general adult population ^15^.

### Decision-analytic modelling

The second proposed economic evaluation will comprise a lifetime Markov cohort model with a 6-month cycle length. This will be constructed if the optimal dose of anti-TNF is found to be effective in controlling the disease progression (in terms of showing a statistically significant difference between the anti-TNF or no treatment groups based on the primary or secondary outcomes) at 12 months follow-up ^2^. A long-term model is needed as this treatment is expected to reduce the proportion of patients progressing to late stage DD. Disease progression of the patients in the trial will be tracked using CRFs which record the nodule size on ultrasound scan, range of motion of affected digit and patient reported outcome measures. A Markov model approach was chosen as the events occur repeatedly over time.

**Figure 1.**
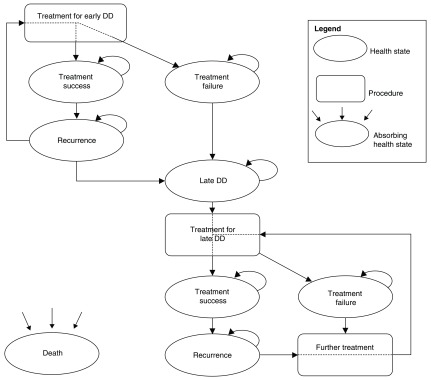
Schematic representation of the preliminary economic model structure.

The preliminary model ( Figure 1) will consist of the following health states: treatment success and failure of early stage DD, recurrence of early stage DD, development of late stage DD, treatment success and failure of late stage DD, recurrence of late stage DD and death. Late stage DD will be defined as flexion deformities of 30 degrees or greater at the metacarpophalangeal or at the proximal interphalangeal joint with a limitation of hand function. Recurrence for early stage DD will be defined as an increase in nodule hardness or size on ultrasound scan or increase of flexion deformity following successful treatment. Recurrence for late stage DD will be defined as the recurrence of contracture of 30 degrees or more in a joint of the digit that was successfully treated to achieve correction to within 5 degrees of neutral ^16^. Treatment success for both early stage DD and late stage DD would be defined as no change or an improvement in nodule hardness or size, or flexion deformity. Treatment failure for both early- and late stage DD will be defined as progression of the disease such that the patient seeks further intervention, usually as a result of deteriorating hand function and/or flexion deformity at the interphalangeal joints of more than 30 degrees. Age and sex-specific all-cause mortality data will be incorporated in the model based on interim UK life tables, which are available from the Office of National Statistics, if there are no published evidence which indicates a difference in mortality between patients with DD and equivalent controls.

Treatments for early stage DD would be the interventions in the RIDD trial (anti-TNF versus usual care) while those for late stage DD would be the interventions stated in the study conducted by Brazelli *et al.*, ^17^ namely collagenase clostridium histolyticum, percutaneous needle fasciotomy and limited fasciectomy.

Model parameters such as the costs, health utilities and transition probabilities for early stage DD in the first 12 months will be informed using data from the second part of the RIDD trial. More specifically, the transition probabilities for the early stage DD will be determined from the proportion of patients who moved to the next health stated based on the changes in range-of-motion measurements obtained during the trial. The utilities and costs of patients at each health state will in turn be determined from the EQ-5D-5L and health resource questionnaires administered during the trial. These model parameters for late stage DD will be obtained from 18 months data of the RIDD trial and from published literature as required, including Brazelli *et al.* ^17^ and Gu *et al.* ^15^.

Adverse events from the treatment of early stage DD, if any, will be modelled using data from the RIDD trial while adverse events/complications for treatment of late stage DD will be modelled using published literature, for example the publication by Chen *et al.* ^18^.

### Data analysis

***Inflation and discounting.*** Due to the small trial sample size (n=35) to be recruited from the Netherlands and to simplify the analysis, the same unit costs (in UK Sterling pounds) for resource utilisation in the Netherlands will be assumed as we do not foresee significant differences in the health resource utilisation between UK and the Netherlands. However, if there is a significant difference in relevant cost items such as length of stay, we will adjust them to the average value in the country of interest for the base case analysis (UK). This is in line with the International Society for Pharmacoeconomics and Outcomes Research (ISPOR) guideline which recommends doing multivariable cost regressions to adjust for country effects as one of its recommendations for estimating country-specific costs for multinational studies ^19^. All costs will be revalued for analysis when appropriate and reported to latest UK prices using the NHS hospital & community health services (HCHS) index for health service resources. For lifetime estimates, the costs and QALYs will be discounted at 3.5% according to UK Treasury guidelines ^20^.

***Statistical analysis.*** Analysis will be performed on an intention-to-treat basis. Mean and associated measures of uncertainty of costs for each cost category, as well as for estimated QALYs, will be calculated at each time point within each trial arm. Differences between these means will be calculated and tested for statistically significant differences from zero using parametric t-tests.

***Cost-utility analysis.*** Data collected will be used to calculate the cost and QALY per trial participant over the 18 month time horizon of the trial; the baseline to 12 months trial data will be used in the within-trial CUA, while the 18 months trial data will be used in the long term decision analytic model. In the base case analysis, costs examined from the health system perspective would consist of the direct medical costs and direct nonmedical costs. Using these data, the mean difference in costs and the mean difference in QALYs between the control group (treatment) and the intervention group (anti-TNF) will be estimated to give an incremental cost effectiveness ratio (ICER) and an estimate of incremental net benefit (INB). A cost-effectiveness threshold of £20,000 per additional QALY as recommended by NICE will be used to estimate the cost-effectiveness of the intervention ^21^. An intervention with an ICER below the £20,000 per QALY threshold will generally be considered cost-effective.

***Missing data.*** The nature and pattern of the ‘missing-ness’ will be carefully considered; reasons for missing data will be ascertained and reported if possible and, if necessary, multiple imputation methods will be applied to address the missing data.

***Sensitivity analysis.*** Several deterministic (one-way sensitivity analysis) and a probabilistic sensitivity analysis to explore uncertainties surrounding key parameters in the within-trial economic evaluation (e.g. including societal perspective) and decision-analytic modelling (e.g. transition probabilities) will be undertaken. In order to explore the missing data assumptions, sensitivity analysis will be run on the per-protocol population and multiple imputation method. Cost assumptions in the analysis will also be modified if relevant.

Results from the deterministic sensitivity analysis will be presented in Tornado diagrams in order to compare the relative importance of the parameters. Results from the probabilistic sensitivity analysis will be presented using cost-effectiveness acceptability curve (CEAC) which shows the probability that anti-TNF is cost-effective relative to no treatment across a range of cost-effectiveness thresholds. The CEAC will be generated based on the proportion of bootstrap replicates with positive incremental net benefits ^22, 23^. The probability that anti-TNF is less costly or more effective than no treatment will be based on the proportion of bootstrap replicates that have negative incremental costs or positive incremental health benefits, respectively.

## Discussion

RIDD is the first randomised controlled trial with an economic evaluation conducted among patients with early stage DD; previous studies had utilised decision analytic models (two using expected value decision analytic models and two using Markov models) to estimate the cost-effectiveness in the management of late stage DD ^3^.

Key strengths of the economic evaluation conducted alongside the trial include a comprehensive assessment of health and social care services resource usage and a reliable method for estimating unit costs from published national sources ^24^. Economic evaluation conducted alongside the trial also allows reliable estimates of cost effectiveness to be produced at low marginal cost and a wide range of statistical and econometric tests can be utilised since data will be at an individual level ^25^.

Key limitations of the economic evaluation include response bias and non-response bias due to the nature of the data collection (via patient-reported questionnaires) ^26^. Response bias occurs when there is a systematic difference in the way participants answered such that their answers do not accurately represent their experience, while non-response bias occurs when there is a systematic difference in characteristics between the responders and non-responders ^27^. Furthermore, we are not considering the health system perspective of the Netherlands due to the relatively small number of patients being recruited although we would be adjusting for any country effects in our analysis.

In our view, presenting this methodology paper, which serves as a standard operating procedure, before the end of the trial helps safeguards the transparency and consistency of the steps that should be followed as part of the evaluation ^28^. This in turn will improve the robustness of our evaluation of the health economic data from the RIDD trial.

## Data availability

No data are associated with this article.
