# Supplementary material for: Economic evaluation plan of a randomised controlled trial of intra-nodular injection of anti-TNF and placebo among patients with early Dupuytren’s disease: Repurposing Anti-TNF for Treating Dupuytren’s Disease (RIDD)
Source: Wellcome Open Res. 2019 Feb 12;3:156. Originally published 2018 Dec 7. [Version 2] doi: 10.12688/wellcomeopenres.14936.2 (PMC6354323; doi:10.12688/wellcomeopenres.14936.2)
Supplement: Peer review history

# Peer review: Referee response for version 1

**DOI:** 10.21956/wellcomeopenres.16285.r34551

**Published:** 2019-01-30

**Authors:** Raymond Oppong

**Affiliations:** 1 Arthritis Research UK Primary Care Centre, Research Institute for Primary Care and Health Sciences, Keele University, Keele, UK; 2 Health Economics Unit, Institute of Applied Health Research, University of Birmingham, Birmingham, UK

The aim of this study is to describe the methods for assessing the cost-effectiveness of adalimumab injections compared to placebo for the management of dupuytren’s disease.  The authors propose a within-trial economic evaluation over 12 months and a model-based economic evaluation over a lifetime horizon if the intervention is found to be effective.   

 This protocol is very well written and the methods adequately described. Resource use, cost and health outcome data collection for the economic evaluation have been adequately described.

 A potential limitation of the within-trial economic evaluation is the placebo comparator. Normally, you would want to compare treatments with usual care/practice.

 The paper states on page 3 that “For both analyses, a health system (National Health Service (NHS) and personal social services (PSS)) perspective will be adopted for the base case analysis” A health system can be broader than this depending on how you choose to define it.

 With respect to the costing, even though assume the same unit costs for the UK and Netherlands has been justified in this case, it may be worth exploring other options (e.g. the effect of multicountry costing) in sensitivity analysis.

 The model could also be better described. E.g. the description provided in the paper just mentions treatments for early and late stage DD but does not give an indication of what these treatments are.

 The authors state that a DAM will be conducted if the intervention is found to be effective at 12 months. A better justification should be provided for this.

I have read this submission. I believe that I have an appropriate level of expertise to confirm that it is of an acceptable scientific standard.

# Response

**Published:** 2019-02-08

**Authors:** May Ee Png

**Affiliations:** University of Oxford, UK

We would like to thank both Mrs Belen Corbacho and Dr Raymond Oppong for their supportive reports and helpful comments about our health economics analysis plan (HEAP). We have amended the HEAP manuscript accordingly.

**Comment 1:** A potential limitation of the within-trial economic evaluation is the placebo comparator. Normally, you would want to compare treatments with usual care/practice.

**Response:** As mentioned in our response to Reviewer 1’s Comment 5, we are updated the methodology and relevant text in the abstract and main text to compare anti-TNF with usual care.

**Comment 2: **The paper states on page 3 that “For both analyses, a health system (National Health Service (NHS) and personal social services (PSS)) perspective will be adopted for the base case analysis” A health system can be broader than this depending on how you choose to define it.

**Response:** As defined in our original statement, health system refers to the NHS and PSS. 

 We’ve changed the statement as follows:

*For both analyses, a health system (i.e. National Health Service (NHS) and personal social services (PSS)) perspective will be adopted for the base case analysis…*

**Comment 3:** With respect to the costing, even though assume the same unit costs for the UK and Netherlands has been justified in this case, it may be worth exploring other options (e.g. the effect of multicountry costing) in sensitivity analysis.

**Response: **As mentioned under “Data analysis – Inflation and discounting”, we would estimate this cost according to the ISPOR guideline which recommends performing multivariable cost regressions to adjust for country effects for estimating country-specific costs for multinational studies. Furthermore, as mentioned in the ISPOR guideline, "intercountry differences in population characteristics and treatment patterns are more likely to influence summary measures between countries rather than differences in unit costs". Furthermore, since only a relatively small number of patients will be recruited from Netherlands (n=35) versus at least 138 patients from the UK, it is unlikely that the overall cost results will differ significantly even if other approaches were adopted.

 Reference: Ramsey SD, Willke RJ, Glick H, *et al.* Cost-effectiveness analysis alongside clinical trials II - An ISPOR good research practices task force report. *Value Heal* 2015;18:161–72.

**Comment 4: **The model could also be better described. E.g. the description provided in the paper just mentions treatments for early and late stage DD but does not give an indication of what these treatments are.

**Response: **We have included a paragraph under “Methods – Decision-analytic modelling” as follows:

*Treatments for early stage DD would be the interventions in the RIDD trial (anti-TNF versus placebo) while those for late stage DD would be the interventions stated in the study conducted by Brazelli et al [17], namely collagenase clostridium histolyticum, percutaneous needle fasciotomy and limited fasciectomy.*

**Comment 5: **The authors state that a DAM will be conducted if the intervention is found to be effective at 12 months. A better justification should be provided for this.

**Response:** As detailed in our response to Reviewer 1’s Comment 1, we have updated the methodology for better clarification.

# Peer review: Referee response for version 1

**DOI:** 10.21956/wellcomeopenres.16285.r34411

**Published:** 2019-01-14

**Authors:** Belen Corbacho

**Affiliations:** 1 Department of Health Sciences, York Trials Unit, University of York, York, UK

I have no concern about economics methods as they seem robust and according to current best practice for such analyses. The research team has extensive experience in their field of health economics and clinical trials. Therefore the authors place the team in a great position to undertake and informs a timely research question.

 The authors have appropriately justified the need for this study. The economic evaluation plan provides a thorough overview of the cost-effectiveness analysis that will be conducted in the RIDD trial. Nevertheless I have some comments/questions the authors might want to address:

- In addition to the main within-trial economic evaluation (cost per QALY approach in accordance with NICE guidance), the co-applicants also plan to conduct modelling work if deemed appropriate. The authors say that they will only do the model if the treatment is effective. I advise the authors to explain this further (e.g effective on primary outcome statistical significance / primary outcome not statistical significance / effective on secondary outcomes / effective on QoL).
- I would suggest including a section to summarize the review of existing literature. The authors are planning to use a number of economic evaluations, however they give no detail about databases searched, number of relevant evaluations identified, model structure in relevant papers, categories of resource use included in the models etc. They could include a table as Annex to give further details on this.
- The authors should provide the reader with a justification for the need of the long term model. A key point is to clarify what is the treatment expecting to capture, which I would advise to explain further in the text. Is the treatment expected to delay progression to late DD? If this is the case: (i) how quickly this is expected (e.g is the follow-up of the trial enough to capture this?); and (ii) how is this captured? If this is captured in the RIDD trial this should be listed (i.e percentage of patients that become late DD). If this is not captured in the trial, is there any relevant study that relates the primary or secondary outcome to late DD? I think this is a critical point, because if treatment is not expected to delay progression then it’s all about symptomatic management, and if this is the case there might be no need for such a sophisticated model.

- Similarly, I would suggest to provide more detail on the statistical analysis which will inform the model: how it is planned to estimate transition probabilities, utilities of health states and costs. My main concern is how the authors are planning to implement the model, given that the trial has 138 patients to inform transitions. It might be helpful to justify the numbers in the analysis plan; and consider the possibility of simplifying the model structure according to these numbers.

- Regarding utilities, I would like to see further details about how they are getting the utilities from the trial. For example, details about the statistical model that will be used to define eq5d given the health state. Similarly, more detail should be given to how costs related to health stated will be estimated (e.g sources).

- In relation to direct costs, I am not sure I understand the decision of excluding man power. In clinical practice it won´t be about the new drug vs placebo but the new drug vs current treatment. Therefore, I don’t think excluding man power is correct, as cost-effectiveness is about clinical practice, and in clinical practice there will be staff costs related to the treatment, even if outpatient visit is only £80, this should also be considered. 

I have read this submission. I believe that I have an appropriate level of expertise to confirm that it is of an acceptable scientific standard.

# Response

**Published:** 2019-02-08

**Authors:** May Ee Png

**Affiliations:** University of Oxford, UK

We would like to thank both Mrs Belen Corbacho and Dr Raymond Oppong for their supportive reports and helpful comments about our health economics analysis plan (HEAP). We have amended the HEAP manuscript accordingly.

**Comment 1:** In addition to the main within-trial economic evaluation (cost per QALY approach in accordance with NICE guidance), the co-applicants also plan to conduct modelling work if deemed appropriate. The authors say that they will only do the model if the treatment is effective. I advise the authors to explain this further (e.g effective on primary outcome statistical significance / primary outcome not statistical significance / effective on secondary outcomes / effective on QoL).

**Response:** As mentioned in the “Decision-analytic modelling” section, the modelling will be performed if the trial shows that anti-TNF injection is found to be effective in controlling the disease progression at 12 months follow-up (which is the primary outcome). The text has been changed as follows in order to clarify this point:

*The second proposed economic evaluation will comprise a lifetime Markov cohort model with a 6-month cycle length. This will be constructed if the optimal dose of anti-TNF is found to be effective in controlling the disease progression in terms of showing a statistically significant differences between the anti-TNF or no treatment groups based on the primary or secondary outcomes at 12 months follow-up.*

**Comment 2:** I would suggest including a section to summarize the review of existing literature. The authors are planning to use a number of economic evaluations, however they give no detail about databases searched, number of relevant evaluations identified, model structure in relevant papers, categories of resource use included in the models etc. They could include a table as Annex to give further details on this.

**Response:** We have already described the current state of the value for money of competing alternatives to treat and manage Dupuytren’s disease in a recent literature review (Dritsaki, 2018); hence we have updated our Introduction to reflect this:

*However, a recent systematic review published by the trial team [3] identified four studies, all of which synthesised evidence from various sources, rather than a single randomised controlled trial, and reported the cost-effectiveness of collagenase clostridium histolyticum injection, percutaneous needle fasciotomy or limited fasciectomy among patients with late stage DD.*

 Reference: Dritsaki M, Rivero-Arias O, Gray A, et al. What do we know about managing Dupuytren’s disease cost-effectively? *BMC Musculoskelet Disord* 2018;19:34. doi:10.1186/s12891-018-1949-2

**Comment 3:** The authors should provide the reader with a justification for the need of the long term model. A key point is to clarify what is the treatment expecting to capture, which I would advise to explain further in the text. Is the treatment expected to delay progression to late DD? If this is the case: (i) how quickly this is expected (e.g is the follow-up of the trial enough to capture this?); and (ii) how is this captured? If this is captured in the RIDD trial this should be listed (i.e percentage of patients that become late DD). If this is not captured in the trial, is there any relevant study that relates the primary or secondary outcome to late DD? I think this is a critical point, because if treatment is not expected to delay progression then it’s all about symptomatic management, and if this is the case there might be no need for such a sophisticated model.

**Response: **The treatment is expected to reduce the proportion of patients progressing to late-stage DD. Regarding (i), since we are only recruiting patients with a clear history of disease progression, we anticipate that at least some will progress to the late disease stage. For (ii), disease progression will be tracked using case report forms (CRFs) which record the nodule hardness, nodule size via ultrasound imaging, range of motion of affected digit and patient reported outcome measures.

 The following sentence has been added to the “Methods – Decision-analytic modelling” section:

*A long-term model is needed as this treatment is expected to reduce the proportion of patients progressing to late stage DD. *Disease progression of the patients in the trial will be tracked using CRFs which record nodule hardness, the nodule size on ultrasound scan, range of motion of affected digit and patient reported outcome measures.

**Comment 4:** Similarly, I would suggest to provide more detail on the statistical analysis which will inform the model: how it is planned to estimate transition probabilities, utilities of health states and costs. My main concern is how the authors are planning to implement the model, given that the trial has 138 patients to inform transitions. It might be helpful to justify the numbers in the analysis plan; and consider the possibility of simplifying the model structure according to these numbers.

 Regarding utilities, I would like to see further details about how they are getting the utilities from the trial. For example, details about the statistical model that will be used to define eq5d given the health state. Similarly, more detail should be given to how costs related to health stated will be estimated (e.g. sources).

**Response:** The “Methods – Decision-analytic modelling” section has been updated as follows:

*Model parameters such as the costs, health utilities and transition probabilities for early stage DD in the first 12 months will be informed using data from the second part of the RIDD trial. More specifically, the transition probabilities for the early stage DD will be determined from the proportion of patients who moved to the next health stated based on the changes in range-of-motion measurements obtained during the trial. The utilities and costs of patients at each health state will in turn be obtained from the EQ-5D-5L and health resource questionnaires administered during the trial. These model parameters for late stage DD will be obtained from 18 months data of the RIDD trial and from published literature as required, including Brazelli et al. [17] and Gu et al. [15].*

**Comment 5:** In relation to direct costs, I am not sure I understand the decision of excluding man power. In clinical practice it won´t be about the new drug vs placebo but the new drug vs current treatment. Therefore, I don’t think excluding man power is correct, as cost-effectiveness is about clinical practice, and in clinical practice there will be staff costs related to the treatment, even if outpatient visit is only £80, this should also be considered.

**Response:** We have updated the methodology (in the “Methods – Direct medical costs related to trial” section) to include the cost of manpower as suggested by the reviewer. The following has been added:

*Since the usual care is no treatment, the cost of manpower will be included for the anti-TNF group only. Manpower cost will be estimated by assuming that the injection will be administered by a medically qualified clinician at consultant level in the outpatient setting.*

 To make things clearer for readers without health economics background in trials, we have also added the following at the beginning of the Methods section:

*No treatment instead of placebo will be used as the comparator. In the RIDD trial placebo is being administered as an experimental control and not used in routine care for patients with early stage DD.*
